# ISG15 silencing increases cisplatin resistance via activating p53-mediated cell DNA repair

**DOI:** 10.18632/oncotarget.22488

**Published:** 2017-11-18

**Authors:** Yi Huo, Zhaoyun Zong, Qingtao Wang, Zhenyu Zhang, Haiteng Deng

**Affiliations:** ^1^ MOE Key Laboratory of Bioinformatics, School of Life Sciences, Tsinghua University, Beijing, China; ^2^ Beijing Chaoyang Hospital Affiliated to Capital Medical University, Beijing, China

**Keywords:** cancer cells, drug-resistance, proteomics, ISG15, cisplatin

## Abstract

Tumor cells frequently evolved resistance to cisplatin that greatly compromises the efficacy of chemotherapy. Identification of the mechanisms underlying drug resistance is important for developing new therapeutic approaches. ISG15 is found to be elevated in many human carcinomas and cancer cell lines. Here, we identified that the expressions of ISG15 and ISG15-conjugating system were downregulated in drug resistant A549/DDP cells compared to drug sensitive A549 cells. Silencing of ISG15 robustly elevated the resistance to cisplatin, suggesting ISG15 plays an important role in cisplatin resistance. Quantitative proteomics identified 1296 differentially expressed proteins between the control and ISG15 knockdown cells, showing that ISG15 silencing upregulated proteins in p53 pathway, adherens junction and nucleotide excision repair (NER) pathway. We also found that ISG15 silencing induced cell cycle arrest through stabilizing p53 and increasing HnRNP K expression, which allowed the prolonged time for cells to repair cisplatin-damaged DNA. Taken together, we proved that ISG15 downregulation activated the DNA damage/repair pathway to enhance cisplatin resistance in tumor cells.

## INTRODUCTION

ISG15, interferon-stimulated gene 15, is the first reported member of the ubiquitin-like protein (UBL) superfamily [1]. ISG15 was extensively studied in innate immune response to viral infection [2–4]. It also functions in diverse cellular pathways in the form of ISGylation, an ubiquitin-like modification whereby ISG15 is conjugated to target proteins through a C-terminal diglycine (LRLRGG) motif [5]. Like ubiquitination, the coupling of ISG15 to its targets involves the ISG15-activating E1-like protein (UBE1L) [6] and ISG15-conjugating E2 enzyme (UbcH8) [7, 8]. EFP and Herc5 are reported to be the E3 ligases for ISGylation [9, 10]. Deconjugation of ISG15 from target proteins is accomplished by UBP43 [11]. Notably, type I IFNs upregulate the expression of enzymes in ISGylation system. A large number of proteins have been identified as the targets of ISGylation by proteomics studies [12–14]. However, only a small set of candidates have been verified and functionally studied [15, 16]. The functions of ISG15 and ISGylation are proposed to be diverse and cell-type/tissue-specific [17]. ISGylation negatively regulates the ubiquitin-proteasome degradation by directly interfering with polyubiquitination [18]. Elevated expressions of both ISG15 and ISG15-conjugating system were found in tumors of different histological origin [19–24]. Studies showed that chemotherapeutic drugs induced upregulation of ISG15 and ISG15-conjugating system, suggesting that ISG15 and ISGylation inhibited oncogenesis [25, 26]. Dysregulated expression of ISG15 was linked to resistance to radiation and chemotherapeutics in tumor cells, suggesting ISG15 was a biomarker for drug sensitivity [27–29].

Cisplatin has been widely used for the past decades to treat testicular, ovarian, cervical, head and neck, non-small-cell lung and melanoma cancer [38, 39]. However, the treatment efficacy is compromised by the high rate of acquiring resistance to currently available drugs [40–42]. Therefore, it is important to dissect the mechanism underlying cisplatin resistance. Cisplatin treatment causes DNA damage that induces cell death. Under DNA damage, the transcription factor p53 is activated by ATM, ATR, and DNA-PK which in turn regulates the downstream effector proteins and activates DNA damage/repair pathway, which determines the cell fate [30–32]. The major downstream event of p53 activation is the cell cycle arrest at G1/S or G2/M transition stages for the repair of damaged DNA, in which the effector proteins such as p21 WAF/CIP induces G1/S arrest [33] while effector proteins such as 14-3-3 sigma, GADD45, and Reprimo are responsible for G2/M arrest [34]. Previous studies showed that p53 mutation decreased the repair efficiency of cisplatin-damaged DNA and reduced cisplatin resistance [35]; And the nucleotide excision repair (NER) system was responsible for repairing cisplatin-induced DNA damage [36, 37]. Upregulation of the DNA repair protein ERCC1 in NER pathway promotes cisplatin resistance [38], whereas tumor cells become sensitive to cisplatin after being treated with the inhibitor of protein PCNA which is the auxiliary protein of DNA polymerase δ [39].

However, less is known whether expressions of ISG15 and ISGylation-related proteins play a role in cisplatin resistance. In the present study, we identified that ISG15 was downregulated in cisplatin-resistance cells and IGS15 silencing increased the cisplatin resistance. Differentially expressed proteins between the control cells and the ISG15 knockdown cells were identified revealing that ISG15 knockdown upregulated proteins associated with p53 pathway, adherens junction and DNA repair. We demonstrated that ISG15 silencing enhanced the p53 stability and upregulated HnRNP K. Furthermore, hnRNP K silencing decreased the cisplatin resistance. Our data proposed a new mechanism underlying ISG15-mediated cisplatin resistance.

## RESULTS

### ISG15 and ISGylation associated proteins are downregulated in cisplatin-resistant cells

**Figure 1:**
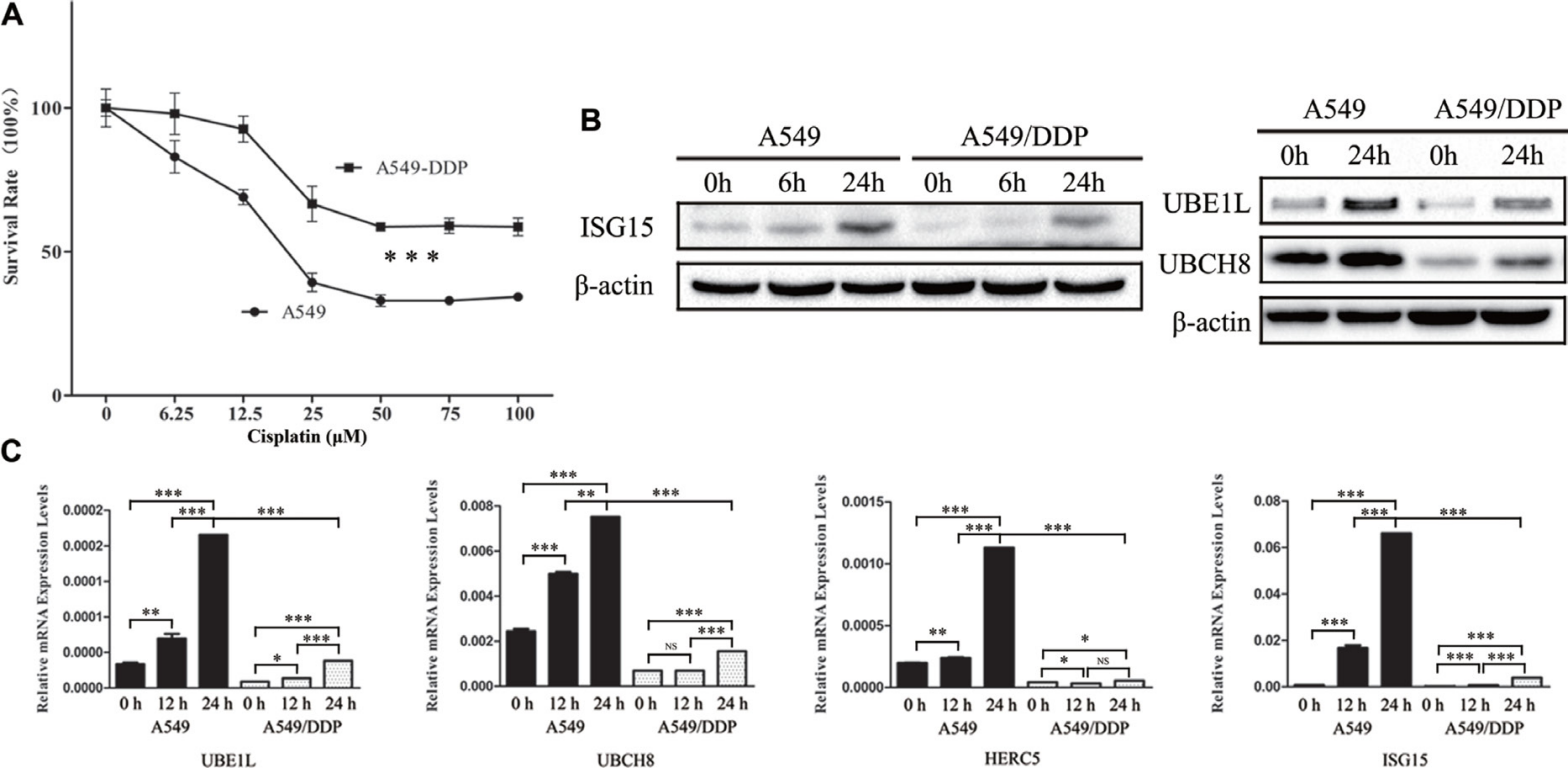
Downregulation of ISG15 and ISGylation-related proteins in cisplatin resistant cells (**A**) Survival rates of A549 and A549/DDP cells treated with different concentration of cisplatin for 24 h. (**B**) Western blotting images of UBE1L, UBCH8 and ISG15 in A549 and A549/DDP cells treated with cisplatin for 0 h, 6 h and 24 h. (**C**) Graphical representation of mRNA expressions levels of UBE1L, UBCH8, HERC5 and ISG15 in A549 and A549/DDP cells treated with cisplatin for 0 h, 12 h and 24 h. All the results show the means of three independent experiments. Error bars indicate SEM. Data were analyzed using Student's *t*-test. ^*^p < 0.05, ^**^p < 0.01 and ^***^p < 0.001.

A549 cells are human alveolar basal epithelial adenocarcinomic cells. A549/DDP cells are cisplatin resistant cells derived from A549 cells that display the higher resistance to cisplatin than the A549 cells (Figure 1A). In the presence of cisplatin, the expressions of ISG15, UBE1L, and UBCH8 were analyzed by western blotting, showing that they were downregulated in A549/DDP cells (Figure 1B). Similarily, the mRNA levels of *ISG15*, *UBE1L*, *UBCH8* and *HERC5* were also lower in A549/DDP cells than those in A549 cells (Figure 1C). This indicates that ISG15 plays a role in cisplatin resistance.

### ISG15 silencing enhanced cisplatin resistance in multiple cell lines

**Figure 2:**
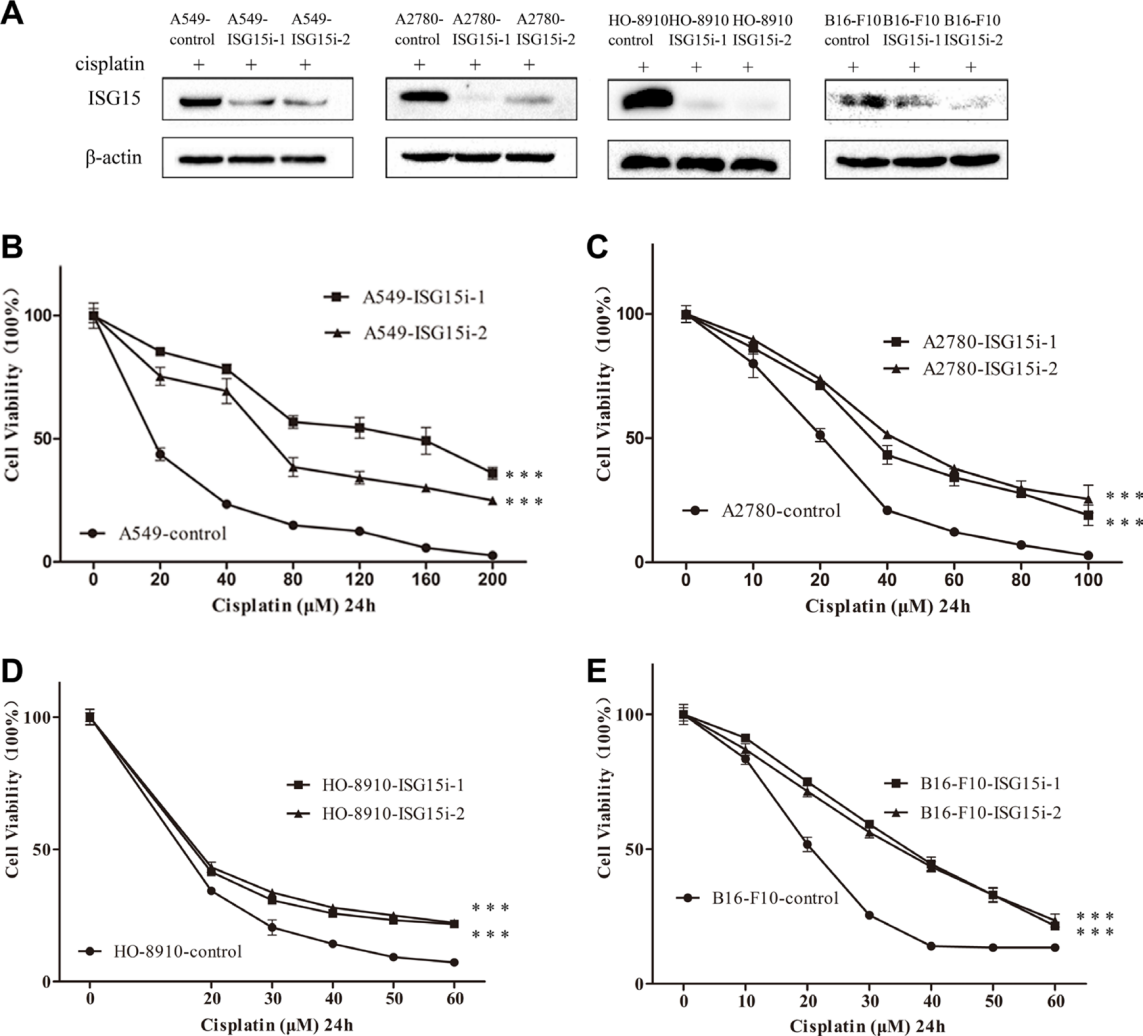
ISG15 silencing increased cisplatin resistance (**A**) Western blotting analysis confirming that ISG15 was downregulated in A549-ISG15i, A2780-ISG15i, HO-8910-ISG15i and B16-F10-ISG15i cells as compared to the control cells. ISG15i-1 and ISG15i-2 stand for the two ISG15 knockdown cell lines using different shRNA. (**B**–**E**) Survival rates of A549-ISG15i, A2780-ISG15i, HO-8910-ISG15i and B16-F10-ISG15i cells and their control cells treated with different concentrations of cisplatin for 24 h. All the results show the means of three independent experiments. Error bars indicate SEM. Data were analyzed using Student's *t*-test. ^*^p < 0.05, ^**^p < 0.01 and ^***^p < 0.001.

To study the role of ISG15 in drug resistance, we silenced ISG15 in A549, A2780, HO-8910, and B16-F10 cell lines (Figure 2A). Two different ISG15-directed shRNAs were used respectively to establish ISG15 silenced cell lines which were referred to as ISG15i-1 and ISG15i-2 cell lines, while the non-target scramble shRNA was used to establish the knockdown control cell line. The survival rates of ISG15 knockdown cells treated with different concentrations of cisplatin for 24 h were determined and compared with those of the respective control cells, showing that ISG15 knockdown robustly increased cell resistance to cisplatin (Figure 2B–2E). The corresponding IC50 values were presented in Supplementary Table 1.

### Identification of differentially expressed proteins between the control and ISG15 knockdown cells

**Figure 3:**
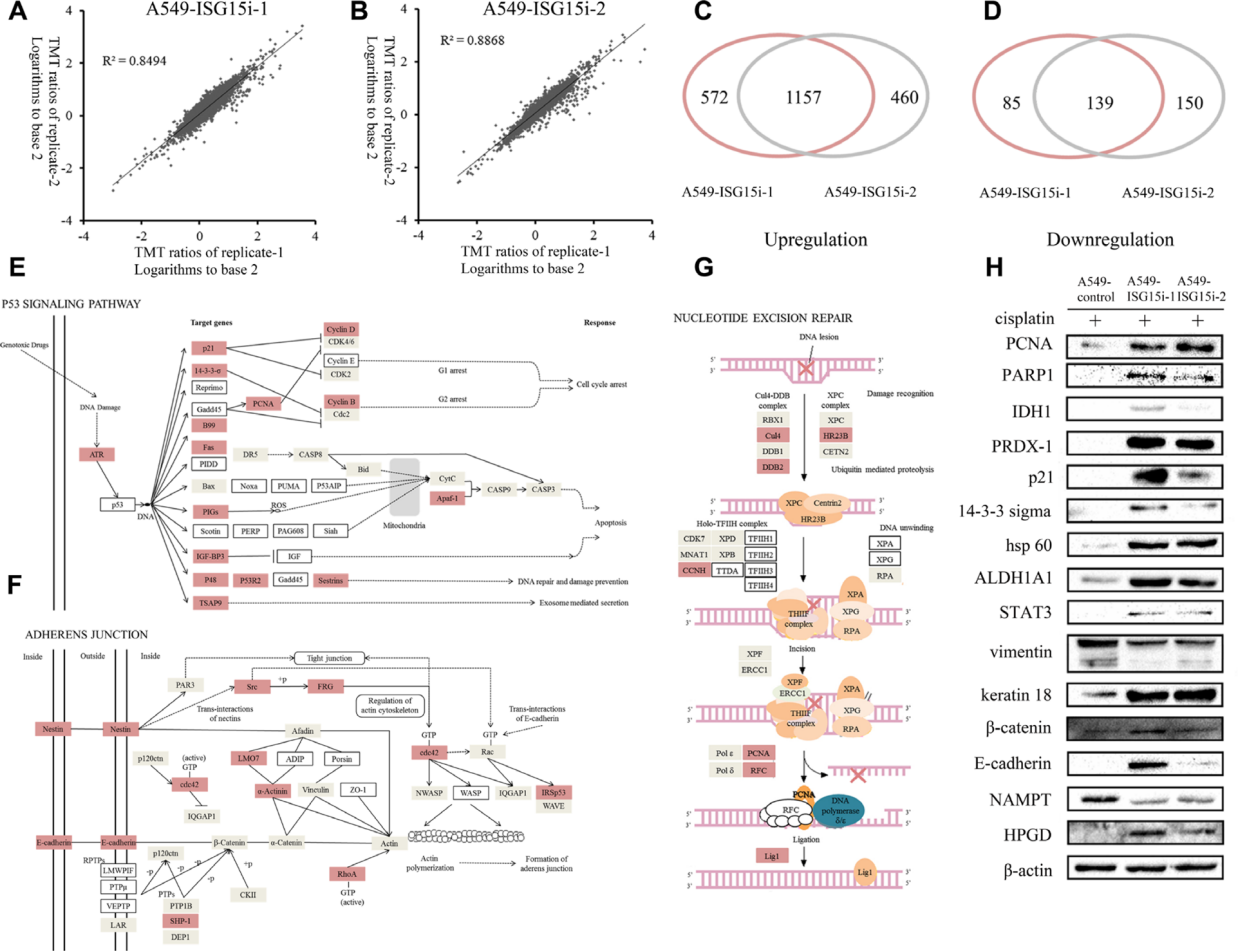
Analysis of differentially expressed proteins between A549-ISG15i-1, A549-ISG15i-2 and control cells (**A**–**B**) Comparison of the TMT ratio between two technical replicates in A549-ISG15i-1 and A549-ISG15i-2 cells. Correlation coefficient (R^2^) is indicated in the plot. (**C**) A diagram showing the number of upregulated proteins identified from A549-ISG15i-1 and A549-ISG15i-2 cells in two biological replicates. (**D**) A diagram showing the number of common downregulated proteins identified from A549-ISG15i-1 and A549-ISG15i-2 cells in two biological replicates. (**E**–**G**) the p53 signaling pathway, adherens junction and nucleotide excision repair pathway were activated based on the pathway analysis of differentially expressed proteins between A549-ISG15i and control cells with KEGG (http://www.kegg.jp/kegg/pathway.html). (**H**) Western blotting images of selected differentially expressed proteins between A549-ISG15i and control cell.

To understand the ISG15-silencing mediated drug resistance, differentially expressed proteins between A549-ISG15i and control cells were identified by a proteomic analysis, in which 9202 proteins were identified. Excluding proteins with score less than 5 or with only one unique peptide matched, 5612 highly confidently identified proteins were used for the further analysis. The logarithms of the TMT ratios of the identified proteins to the base 2 were compared between two technical replicates by a double-logarithmic plot (Figure 3A–3B). Two technical replicates of TMT ratios were highly correlated, suggesting that the proteomics analysis workflow possessed the high technical reproducibility. Based on the mean value of TMT ratios in two technical replicate (>1.4 or <0.7), the two biological replicates shared a significant overlap for the upregulated and downregulated proteins, respectively (Figure 3C–3D). Thus, we identified 1157 upregulated proteins (Supplementary Table 2) and 139 downregulated proteins (Supplementary Table 3) between A549-ISG15i and the control cells.

The differentially expressed proteins were analyzed and their associated pathways were mapped via KEGG, showing p53 signaling pathway, nucleotide excision repair and adherents junction were upregulated in A549-ISG15i cells as compared with control cells (Figure 3E–3G). All figures were derived from the original figures in KEGG. Red-coded blocks represented the upregulated proteins, while the grey-coded ones represented proteins whose expressions were not changed in the proteomic analysis. Proteins which were not found in the proteomic data were marked with the white blocks. Figure 3E showed that ATR and most p53 targeted genes were upregulated in A549-ISG15i cells, indicating ISG15-silencing activated the p53 pathway. Meanwhile, we identified that most upregulated proteins were associated with cell cycle arrest and DNA repair and damage prevention, but not apoptosis, indicating that the p53 pathway activation resulted in cell cycle arrest and DNA repair in A549-ISG15i cells. Especially, proteins regulating nucleotide excision repair were upregulated (Figure 3G). Additionally, we identified other upregulated proteins associated with DNA repair in A549-ISG15i cells including MGMT, SMARCAD1, PSME4, ALKBH1, MCM5, XRCC1, TOP3A, TOP2A, TOPBP1, TOP2B (Supplementary Table 2). These results demonstrated that ISG15-knockdown enhanced the DNA repair. Proteins in adherens junction were upregulated (Figure 3F), showing that ISG15 silencing promoted cell-cell adhesion. The changes in 15 differentially expressed proteins were verified by western blotting, including p21, 14-3-3 sigma, vimentin, keratin 18, β-catenin, E-cadherin, PCNA, PARP 1, IDH1, PRDX-1, hsp60, STAT3, ALDH1A1, NAMPT, and HPGD, confirming the quantification results obtained from the proteomics analysis (Figure 3H). We further identified that PCNA in NER pathway was also upregulated in other ISG15 knockdown cells including A549/DDP, A2780, and HO-8910 cells (Supplementary Figure 1).

### ISG15 knockdown induced cell cycle arrest

**Figure 4:**
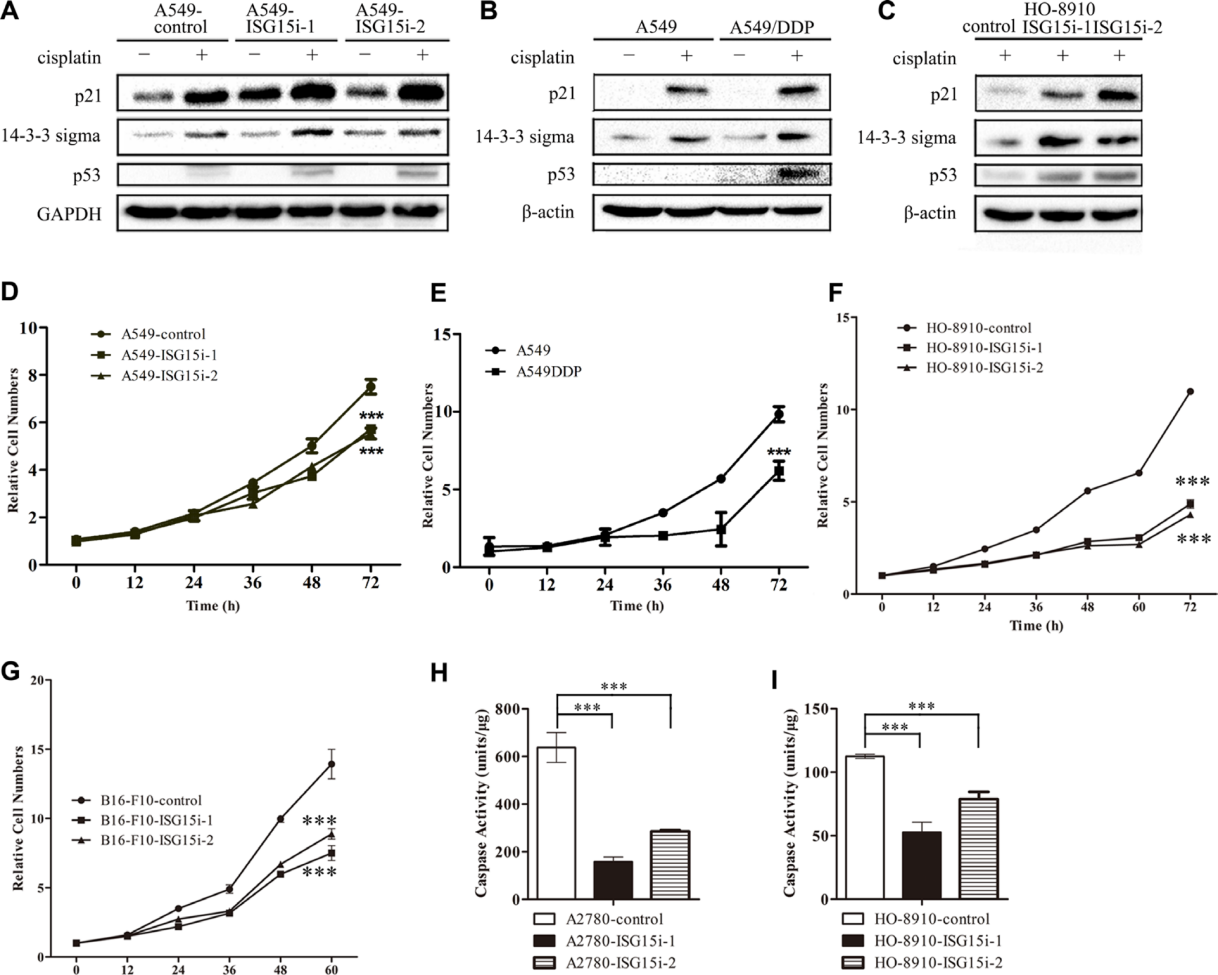
ISG15 knockdown induced cell cycle arrest (**A**–**C**) Western blotting images of p21, 14-3-3 sigma and p53 in A549-ISG15i cells, A549/DDP cells, HO-8910-ISG15i cells as compared with the respective control cells. (**D**–**G**) Growth curves of A549/DDP, A549-ISG15i, HO-8910-ISG15i and B16-F10-ISG15i cells and their respective control cells. (**H**-**I**) Caspase-3 activity analysis in HO-8910-ISG15i cells and A2780-ISG15i cells as compared with the control cells treated with 20 μM cisplatin for 24 h. All the results show the means of three independent experiments. Error bars indicate SEM. Data were analyzed using Student's *t*-test. ^*^p < 0.05, ^**^p < 0.01 and ^***^p < 0.001.

Among differentially expressed proteins between the control and A549-ISG15i cells, the expressions of p21 and 14-3-3 sigma were robustly increased in A549-ISG15i cells in the presence of cisplatin (Figure 3H and Supplementary Table 2). In the present study, we also found that p21, 14-3-3 sigma, and p53 were upregulated in response to cisplatin treatment (Figure 4A–4B), in consistent with previous studies showing that DNA damage upregulated these three proteins leading to cell cycle arrest. However, p53 was not detected in untreated cells by western blott analysis. To determine the effect of ISG15 silencing on p53 expression, qRT-PCR assay was performed to detect changes in p53 mRNA levels, revealing p53 mRNA levels were increased in ISG15 knockdown cells as compared to control cells (Supplementary Figure 2A). Then, we analyzed cell cycle in A549/A549-ISG15i and HO-8910/HO-8910-ISG15i cells by flow cytometry, showing that ISG15 knockdown significantly increased the percentage of cells in the G1 phase (Supplementary Figure 2B). In the ISG15 knockdown cells, we revealed that ISG15 silencing further aggravated upregulation of p21, 14-3-3 sigma, and p53 as confirmed by western blotting in A549 and HO-8910 cells (Figure 4A, 4C), indicating that ISG15 silencing enhanced p53-dependent cell cycle arrest. Consequently, ISG15 knockdown in different cells including A549, HO-8910, and B16-F10 cells reduced cell proliferation and growth (Figure 4D–4G).

To examine whether ISG15 silencing led to apoptosis, we detected the activity of caspase-3 in ISG15 knockdown and control cells, showing that the caspase-3 activity was much lower in ISG15 knockdown cells than that in the control cells in the presence of cisplatin (Figure 4H–4I and Supplementary Figure 2C). These results also demonstrated that ISG15 silencing enhanced cells’ resistance to cisplatin treatment by decreasing the caspase activity. Taken together, it suggests that ISG15 silencing induces the cell cycle arrest and inhibits apoptosis.

### HnRNP K upregulation enhanced the p53/DNA repair pathway and cisplatin resistance in ISG15 knockdown cells

**Figure 5:**
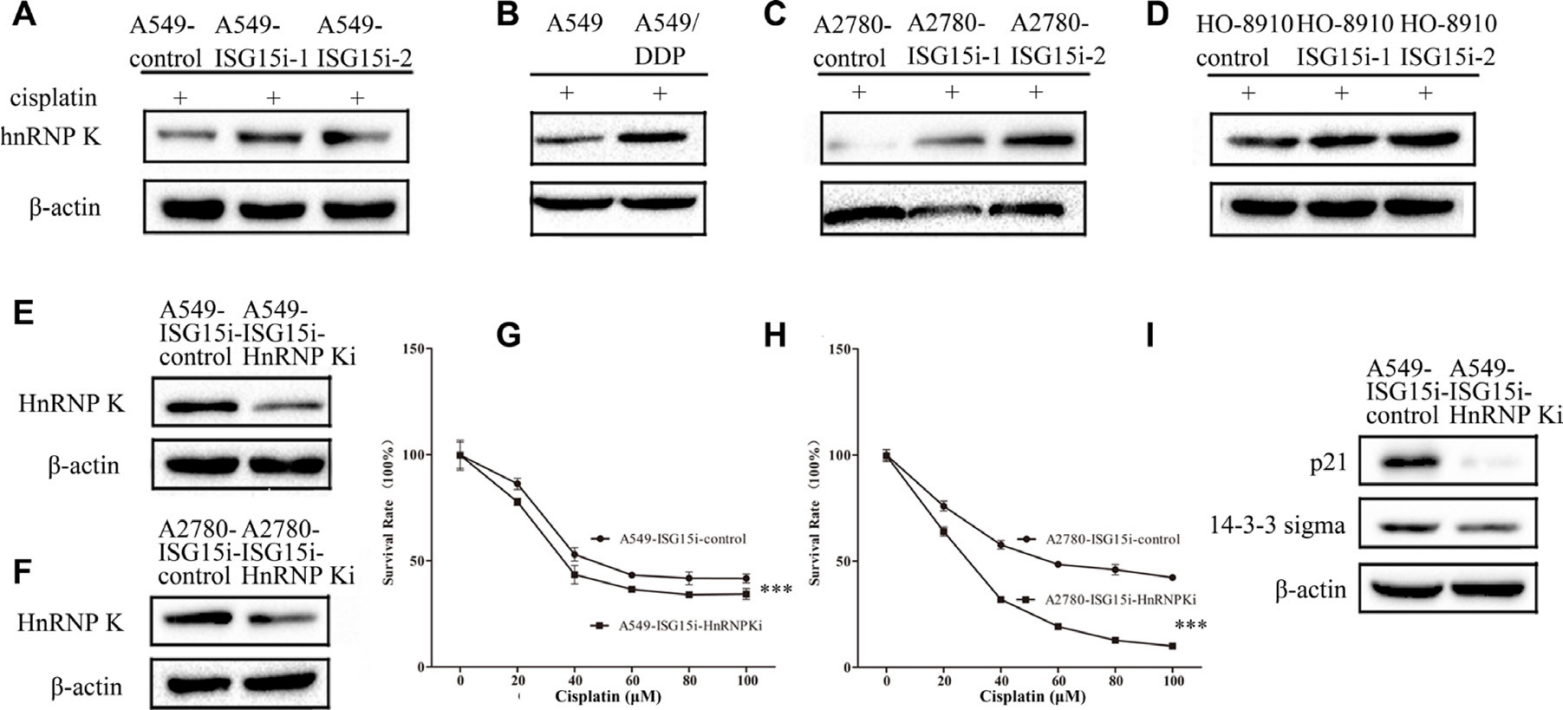
ISG15 silencing upregulated HnRNP K to enhance cisplatin resistance in ISG15 knockdown cells (**A**–**D**) Western blotting images of HnRNP K in A549-ISG15i cells, A2780-ISG15i cells, HO-8910-ISG15i cells and A549/DDP cells compared with the control cells. (**E**–**F**) Western blotting analysis confirming that the expression of HnRNP K was downregulated in A549-ISG15i-HnRNP Ki cells and A2780-ISG15i-HnRNP Ki cells as compared to control cells. (**G**–**H**) Survival rates of A549-ISG15i-HnRNP Ki cells and A2780-ISG15i-HnRNP Ki cells and their control cells treated with different concentrations of cisplatin for 24 h. (**I**) Western blotting images of p21 and 14-3-3 sigma in A549- ISG15i-HnRNP Ki cells as compared with the control cells. All the results show the means of three independent experiments. Error bars indicate SEM. Data were analyzed using Student's *t*-test. ^*^p < 0.05, ^**^p < 0.01 and ^***^p < 0.001.

Proteomics analysis showed that HnRNP K (Heterogeneous nuclear ribonucleoprotein K), a transcriptional coactivator of p53, was upregulated in A549-ISG15i cells in the presence of cisplatin, as confirmed by western blotting (Figure 5A and Supplementary Table 2). The ISG15 silencing mediated HnRNP K upregulation was also identified in other ISG15 knockdown cells (Figure 5C–5D). More importantly, we found that HnRNP K expression was higher in A549/DDP cells than that in A549 cells, in consistent with the finding that ISG15 was downregulated in A549/DPP cells (Figure 5B). These results suggest that HnRNP K plays an important role in drug resistance.

To further study the function of HnRNP K in drug resistance, we silenced HnRNP K in A549-ISG15i-1 and A2780-ISG15i-1 cells (Figure 5E–5F). We revealed that HnRNP K silencing decreased drug resistance in these cells (Figure 5G–5H) while western blotting analysis showed that HnRNP K silencing decreased the expression of p21 and 14-3-3 sigma in HnRNP K knockdown cells as compared to the control cells (Figure 5I), indicating that ISG15 silencing induced HnRNP K upregulation, which activated the p53 pathway.

## DISCUSSION

Understanding the mechanisms underlying drug resistance is important for developing new tumor treatment strategies. In the presence of cisplatin, ISG15 and proteins in ISG15 conjugating system were downregulated in A549/DDP cells as compared to A549 cells, proposing that ISG15 played an important role in cisplatin resistance. This was confirmed by the facts that ISG15 silencing in A549, A2780, HO-8910, and B16-F10 cells resulted in increased cisplatin resistance.

ISG15 knockdown activated adherens junction pathway in A549 cells (Figure 3F), resulting in vimentin downregulation and upregulation of keratin 18, E-cadherin and β-catenin (Figure 3H). This indicates that ISG15 silencing promotes the mesenchymal-epithelial transition (MET) process. ISG15 has been shown to disrupt cytoskeletal architecture, to promote motility, and to trigger metastasis [40–42], which is consistent with our results. However, further studies are needed to determine whether ISG15 silencing activated adherens junction plays a role in drug resistance.

Our results showed that cisplatin treatment upregulated p53 and two effector proteins p21 and 14-3-3 sigma (Figure 4A–4C). Similarily, the expressions of p53, p21, and 14-3-3 sigma were increased in ISG15 knockdown cells (Figure 4A–4C). Previous studies revealed that p53 was a known substrate of ISG15 and p53 ISGylation led to its degradation, whereas ISG15 silencing increased p53 stability [43, 44]. It has been known that p53 plays an essential role in cell cycle arrest, DNA repair and apoptosis. Upregulation in p21 and 14-3-3 sigma expression induces cell cycle and growth arrest upon DNA damage, which provides prolonged time for repairing the damaged DNA. When the damaged DNA is repaired, cells would survive, otherwise, they proceed to apoptosis [45]. In the present study, we found that the activity of caspase 3 was lower in ISG15i cells in the presence of cisplatin, suggesting ISG15 silencing suppressed apoptosis pathway. Furthermore, ISG15 silencing upregulated proteins in NER pathway, especially PCNA and the p53 coactivator HnRNP K. Silencing HnRNP K in ISG15i cells decreased the drug resistance. Taken together, ISG15 silencing enhances the p53 stability and increases HnRNP K expression, which in turn induces p53-dependent cell cycle arrest and allows the prolonged period for repairing cisplatin-induced DNA damage. Thus, we identified that ISG15 is a determinant for cisplatin sensitivity/resistance.

## MATERIALS AND METHODS

### Chemicals and reagents

Dulbecco's modified Eagle's medium (DMEM), Roswell Park Memorial Institute (RPMI) 1640 medium, phosphate buffered saline (PBS), penicillin/streptomycin, trypsin/EDTA, fetal bovine serum were purchased from Wisent (Montreal, Canada). Dithiothreitol (DTT) was purchased from Merck (Whitehouse Station, NJ). Iodoacetamide (IAA) was purchased from Sigma (St Louis, MO). Sequencing grade modified trypsin was purchased from Promega (Fitchburg, WI). The TMT labeling kit was purchased from Thermo-Pierce Biotechnology (Rockford, IL).

### Cell culture

Human epithelial lung cancer cell lines A549 and A549/DDP were obtained from the Chinese Academy of Sciences (Shanghai, China) and cells were grown in RPMI 1640 medium supplemented with 10% FBS and 1% penicillin/streptomycin at 37°C in a humidified incubator with 5% CO2. Mouse melanoma cell line B16-F10 and human epithelial ovarian cancer cell lines A2780 and HO-8910 were grown in DMEM medium supplemented with 10% FBS and penicillin (100 U/ml)–streptomycin (100 mg/ml) at 37°C with 5% CO2 in a humidified incubator.

### Construction of ISG15-knockdown and hnRNP K-knockdown cell lines

The sequences of the two ISG15 targeting shRNAs were GCAACGAAUUCCAGGUGUC and GGACAAA UGCGACGAACCU. The sequences of the hnRNP K targeting shRNAs was CGCACAGTATTTGCTGC AGAAC. NCi was non-targeting scrambled control of shRNA. The oligonucleotides were annealed and inserted into the pll3.7 shRNA expression vector to generate shRNA. Cell lines with high effectiveness of inhibition to target genes were established using the same method showed in our previous paper [46].

### Cell cytotoxicity assay

Cells (1 × 10^4^ each) were seeded in 96-well plates and cultured for 16 h followed by cisplatin treatment at different concentrations (0, 20, 40, 60, 80, 100 and 120 μM) in triplicates for 24 h. Cell numbers was assessed by measuring absorbance at 450 nm with the CCK-8 assay. Cell viability was calculated as the percentage of variable cells compared with untreated cells.

### Proteomics analysis

Proteomic analysis was carried out in biological triplicate. Briefly, 20 μM cisplatin treated A549-ISG15i-1, A549-ISG15i-2 and control cells were lysed with 8 M urea in PBS pH 7.4. Equal amount of proteins from each samples (100 μg) were reduced with 1 mM dithiotreitol for 1 h and alkylated with 5.5 mM iodoacetamide for 40 min in the dark. Proteins were digested with sequencing grade modified trypsin for 16 h at 37°C. After being desalted using C18 sep-pak cartridge, peptides were labeled with TMT sixplex labeling reagent according to the manufacture's instruction and combined. Then the TMT-labeled peptides complex was separated by HPLC. The fractions were collected for LC-MS/MS analysis.

The proteomics data have been deposited to the ProteomeXchange Consortium via the PRIDE partner repository with the data set identifier PXD002825. The files now can be accessed with the username ‘ reviewer11713@ebi.ac.uk’ and password ‘igi3e9MG’, and they will be completely available upon the publication of this manuscript. Details about HPLC separation, LC-MS/MS analysis and searching criteria were showed in our previous paper [46].

### Western blotting and qPCR

For western blotting analysis, equal amount of proteins of control or ISG15i cells were denatured at 100°C boiled 5 min with SDS loading buffer. The proteins were transferred to PVDF transfer membrane by electroblotting after SDS-PAGE separation. Membranes were probed with the indicated antibodies overnight at 4°C followed by immunoblotting analysis. β-actin was internal control. The gray intensity analysis of western blotting images was carried out by imageJ software. Total RNA was reversely transcribed using HIScript 1st Strand cDNA Synthesis Kit (Vazyme, Nanjing, China). The primers for quantitative RT-PCR of cDNA were listed in Supplementary Table 4.

### Cell proliferation assay with CCK-8

Cells were seeded in 96-well plates with 2000 cells/well. Cell proliferation rate was determined with the Cell Counting Kit-8 (CCK-8) according to the manufacturer's instructions (Dojindo Laboratories, Kumamoto, Japan). Briefly, CCK-8 reagents were added into wells after cells grew for 0, 12, 24, 36, 48, 72, 84, 96 h respectively. Absorbance at 450 nm was measured 2 h after CCK-8 addition.

### Caspase-3 activity assay

Cells were cultured for 12 h followed by 20 μM cisplatin treatment in triplicates for 24 h. Then proteins were extracted and quantified. 30 μg proteins of control or ISG15i cells were incubated with 10 μl Ac-DEVD-pNA (2mM) for 2 h in 96-well plates. Caspase-3 activity was assessed by measuring absorbance at 405 nm. Caspase-3 activity was calculated according to the standard curve.

### Cell cycle assay

2 × 10^5^ cells were seeded into each well in 6-well cell culture plate and allowed to grow for 12 hours. Then, cells were trypsinized, washed with cold PBS, and then fixed in pre-cooled 70% ethanol at –20°C for 2 hours. After fixation, cells were washed with cold PBS for two times and re-suspended in 100 μl PBS solution containing 250 μg/ml RNase A, 100 μg/ml propidium iodide (PI) and 0.3% TritonX-100 in dark at room temperature for 45 minutes. Then, the solution was transferred to FACStubes and add another 300 μl PBS buffer for flow cytometry analysis. DNA content was analyzed by BD FACSCalibur (Becton, Dickinson and company) and data were analyzed using the software of CELLQuest (Becton, Dickinson and company). Each experiment was performed in triplicate.

### Experimental design and statistical rationale

All experiments were performed in biological triplicates. Statistical analysis was carried out with GraphPad Prism 5.0 software on data related to cell proliferation assay, cell cytotoxicity assay, PRM analysis and qPCR. In the statistical analysis of cell proliferation assay and cell cytotoxicity assay, the points were fitted to a curve and compared. Significant differences in the data were determined by Student's *t*-test. *P* values of < 0.05 were considered significant.

## SUPPLEMENTARY MATERIALS FIGURES AND TABLES






