# Supplementary material for: ISG15 silencing increases cisplatin resistance via activating p53-mediated cell DNA repair
Source: Oncotarget. 2017 Nov 18;8(64):107452–61. doi: 10.18632/oncotarget.22488 (PMC5746079; doi:10.18632/oncotarget.22488)
Supplement: Supplementary file 1 [file oncotarget-08-107452-s001.pdf]

ISG15 silencing increases cisplatin resistance via activating p53-mediated cell DNA repair

SUPPLEMENTARY MATERIALS

Supplementary Table 1: IC50 of different cell type

Cell type	IC50	Cell type	IC50
8910-Nci	13.83	A2780-Nci	20.43
8910-ISGi-1	16.97	A2780-ISGi-1	36.32
8910-ISGi-2	17.74	A2780-ISGi-2	43.37
A549-Nci	15.61	B16-Nci	19.97
A549-ISGi-1	130.1	B16-ISGi-1	34.73
A549-ISGi-2	65.97	B16-ISGi-2	33.33

Supplementary Table 2: Upregulated Proteins in A549-ISG15i-1 and A549-ISG15i-2 cells. See Supplementary_Table_2

Supplementary Table 3: Downregulated Proteins in A549-ISG15i-1 and A549-ISG15i-2 cells. See Supplementary_Table_3

Supplementary Table 4: Primers for qPCR analysis

Gene Name	Primer
UBE1L-F	GATGTCAGAACTACGGGATT
UBE1L-R	GGCGAAAGGCACTACGAG
UBCH8-F	ATGCGAGTGGTGAAGGAG
UBCH8-R	GTGGTAGGGAGGTTGGTC
ISG15-F	GGACAAATGCGACGAACC
ISG15-R	CCCGCTCACTTGCTGCTT
HERC5-F	GAAGCTGCACAGGGTAAA
HERC5-R	GAAGCGTCCACAGTCATT

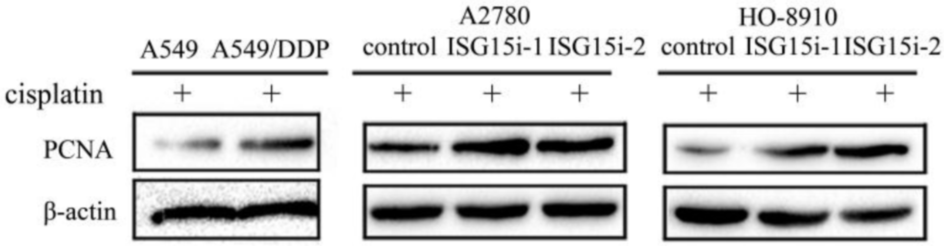

Supplementary Figure 1: Western blotting images of PCNA in A549/DDP cells, A2780-ISG15i cells, and HO-8910-ISG15i cells as compared with relevant control cells.

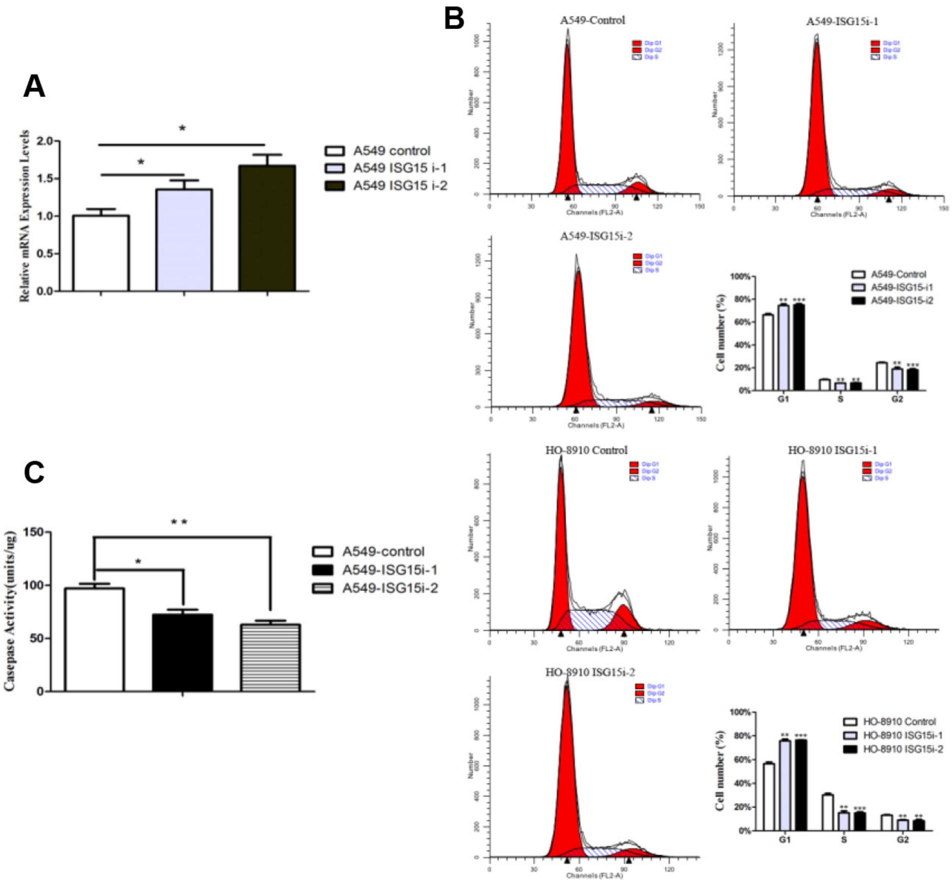

Supplementary Figure 2: (**A**) q-PCR result of p53 expression level without cisplatin treatment in A549 and A549-ISG15i. (**B**) Caspase-3 activity analysis in A549-ISG15i cells as compared with relevant control cells treated with 20 μM cisplatin for 24 h. (**C**) cell cycle arrest analysis of A549/A549-ISG15i and HO-8910/HO-8910-ISG15i cells by flow cytometry. All the results show the means of three independent experiments. Error bars indicate SEM. Data were analyzed using Student's *t* test. $^{*}p < 0.05$, $^{**}p < 0.01$ and $^{***}p < 0.001$.
